# Birthing injustice: unmasking structural racism and the illusion of reconciliation in indigenous perinatal care in Canada, insights from the RESPCCT study

**DOI:** 10.3389/fgwh.2026.1780855

**Published:** 2026-07-01

**Authors:** Wanda Phillips-Beck, Nisha Malhotra, Kathrin Stoll, Elder Roberta Price, Saraswathi Vedam

**Affiliations:** 1 University of Manitoba, Indigenous Research Chair in Nursing, First Nations Health and Social Secretariat of Manitoba, Winnipeg, MB, Canada; 2 Birth Place Lab, Faculty of Medicine, University of British Columbia, Vancouver, BC, Canada; 3 Snuneymuxw and Cowichan Nations, Richmond, BC, Canada

**Keywords:** childbirth, colonial violence, cultural safety, human rights, indigenous health, mistreatment, respectful maternity care, truth and reconciliation commission (TRC)

## Abstract

Indigenous people in Canada continue to experience systemic racism, disrespect, and loss of autonomy within the healthcare system, particularly in the context of pregnancy and childbirth. Despite national commitments to reconciliation and the recognition of Indigenous rights through frameworks such as the Truth and Reconciliation Commission (TRC) and the United Nations Declaration on the Rights of Indigenous Peoples (UNDRIP), the realities of maternity care for Indigenous people are shameful. This study draws on data from a national survey examining respect, mistreatment and autonomy in pregnancy and childbirth care across Canada, with focused analysis of the experiences of Indigenous respondents. The findings reveal persistent racism, lack of culturally safe care, and widespread violations of their rights, particularly informed consent and bodily autonomy. Mistreatment and disrespect were more common among Indigenous participants, participants who had an Indigenous partner, and those aged 25 and younger (*p* < 0.001, Bonferroni-corrected). Through an Indigenous-led lens, this analysis challenges the performative nature of reconciliation in Canadian healthcare system and calls for transformative change rooted in accountability, Indigenous knowledge systems, and in the restoration of birthing sovereignty. These results underscore the urgent need to center Indigenous voices and leadership in reorienting perinatal care policy, practice, and education.

## Introduction

Indigenous Peoples hold inherent rights to health, cultural integrity, and self-determination, as affirmed in the Truth and Reconciliation Commission of Canada Calls to Action, the United Nations Declaration on the Rights of Indigenous Peoples, and the Universal Declaration of Human Rights. Together, these instruments affirm the obligation of healthcare systems to provide culturally safe, trauma-informed, and respectful care for Indigenous People. However, Indigenous Peoples in Canada continue to experience persistent health disparities arising from ongoing colonial structures embedded within healthcare systems. These structures perpetuate racism, stereotyping, and the marginalization of Indigenous knowledge and cultural practices, undermining trust and creating barriers to equitable care (1–3). These inequities are particularly evident in pregnancy and childbirth, where Indigenous families often encounter discrimination, mistrust in the healthcare system, and inadequate integration of Indigenous knowledge and traditions in medical practices (2).

Experiences of discrimination and systemic inequities during pregnancy can exacerbate emotional strain, increasing stress and anxiety among Indigenous women and other marginalized groups. The link between stress and negative health outcomes for both mothers and infants has been well established (4). The emotional burden experienced by Indigenous mothers as a result of racism and discrimination in healthcare settings can increase the risk of conditions such as postpartum depression, with lasting impacts on both maternal and infant health (5). These experiences are not isolated, but reflect well-documented associations between racism, structural inequities, and adverse maternal mental health outcomes (6, 7). A recent study showed that several groups are significantly more likely to report disrespectful care, loss of autonomy, and mistreatment during childbirth in Canada, including young women, those with high pre-pregnancy BMI, insufficient incomes, and women who identify as racialized or Indigenous (8). Experiences of mistreatment during maternity care can also discourage individuals from seeking postnatal healthcare services (9).

Not only do Indigenous women face significant barriers in accessing culturally safe care, but many who live in northern and remote communities in Canada face barriers accessing healthcare services, and are forced to relocate to urban centers for childbirth. This forced medical evacuation disrupts families and communities, leading to emotional and cultural disconnection, financial strain, and dissatisfaction with health care (10–12). In stark contrast, culturally integrated initiatives like the Inuulitsivik Midwifery Program in Nunavik demonstrate the benefits of community-based birthing services. This program successfully reduced evacuation rates, improved maternal and neonatal health outcomes, and empowered communities by reclaiming traditional birthing practices, addressing systemic inequities, and fostering cultural and emotional well-being (13). Evidence suggests that culturally safe and respectful maternity care approaches can lead to fewer negative health outcomes and greater maternal satisfaction, particularly among marginalized populations (14). In this context, models that emphasize continuity of care, such as midwifery-led services, operationalize these principles and have been shown to enhance maternal experience and infant health outcomes (15, 16).

This study aimed to describe experiences of mistreatment and disrespect of respondents who identified as Indigenous to Canada and participated in the national Research Examining the Stories of Pregnancy and Childbearing in Canada Today (RESPCCT) Study. While regional and qualitative studies have documented Indigenous women's experiences of racism, disrespect, and cultural unsafety in maternity care, few studies have used large-scale survey data or examined these issues within a rights-based framework informed by the United Nations Declaration on the Rights of Indigenous Peoples and the Truth and Reconciliation Commission Calls to Action.

In Canada, we acknowledge three constitutionally recognized Indigenous groups: First Nations, Inuit, and Métis. Each have unique cultures, languages, and histories shaped by their specific relationships to the land and the impacts of colonization, making it essential to recognize these differences to address their unique needs and rights effectively. We, however, frame our discussion within the context of the UNDRIP and the TRC Calls to Action, which refer to Aboriginal and Indigenous peoples, and adopt the term “Indigenous” throughout as a unifying term that acknowledges both the diversity and shared experiences of First Nations, Inuit, and Métis communities in Canada.

## Positionality

The authors approach this research from a shared commitment to culturally safe, equitable, and evidence-informed maternity care. The team includes Indigenous and non-Indigenous researchers, clinicians, and social science scholars, whose perspectives are shaped by both professional practice and lived experience. The first author is an Anishinaabe Nurse Scholar embedded within a First Nation community-based organization, and the RESPCCT study and analytic approaches were guided through ceremony and ongoing relational engagement with Elder Price.

Our varied positionalities informed key aspects of the research process, including the framing of research questions, interpretation of findings, and the application of rights-based frameworks. Indigenous perspectives grounded the analysis in relational, community-informed understandings of health and well-being, while non-Indigenous team members engaged in reflexive practice, attending to their roles and responsibilities in supporting, rather than directing, the interpretation and representation of findings. Decision-making processes were collaborative and relational, with attention to accountability, respect for Indigenous knowledge systems, and the ethical responsibilities inherent in research involving Indigenous Peoples. This approach sought to mitigate power imbalances and ensure that the analysis remained grounded in Indigenous priorities and perspectives.

## Methods

The Canadian Institutes of Health Research funded the RESPCCT study, which examined perinatal care experiences in underrepresented communities across Canada using a community-participatory approach. In 2018–19, a diverse Community Steering Council, representing various geographic and identity groups, was convened to guide the project. This Council was integral in overseeing the study, beginning with an environmental scan to identify community-based, non-governmental organization (NGO) partners that serve underrepresented populations and co-developing the survey instrument.

We compare results of Indigenous participants to non-Indigenous participants and also present Indigenous-specific results, to examine perinatal care experiences among Indigenous with different sociodemographic characteristics and contexts of care. Interpretation was facilitated by an Indigenous scholar drawing on a relational worldview that recognizes the interconnectedness of land, identity, kinship, cultural continuity, governance, and well-being, situating the findings within the ongoing impacts of colonization, including racism and structural inequities in healthcare systems.

The Indigenous scholar mapped results to relevant sections of the Truth and Reconciliation Commission Calls to Action, the United Nations Declaration on the Rights of Indigenous Peoples, and the Universal Declaration of Human Rights, to identify potential violations of these commitments. Analytical decisions were made through collaborative and reflexive processes, with Indigenous scholars guiding the interpretation of statistical findings, which were not treated as value neutral.

Indigenous perspectives informed how variables were conceptualized, how patterns of care were interpreted, and how findings were situated within broader social and historical contexts. For example, measures of neglect, lack of consent, and disrespect were understood not solely as indicators of service quality, but as expressions of relational harm and potential violations of Indigenous Peoples' rights. This approach ensured that analyses reflected Indigenous knowledge, priorities, and lived experiences, and that findings were interpreted in relation to the systemic conditions shaping Indigenous Peoples' experiences.

### Survey development and recruitment

A cross-sectional survey incorporating validated patient-reported outcome and experience measures was initially co-developed with service users, researchers, trainees, and multidisciplinary clinicians. A systematic review and global Delphi process informed selection of items that assess adherence to and violations of respectful maternity care (RMC) within the Canadian healthcare context. Following content validation and pilot testing with 60 service users representing different population groups, the data collection began July 2020 and concluded in February 2022. The survey was disseminated online to partners identified during the environmental scan and via networks maintained by the study team, health professional associations and facilitated by 18 Regional Recruitment Community Coordinators, ensuring a comprehensive reach across all Canadian provinces and territories. Ethics approval for this study was obtained from the UBC Behavioral Ethics Board (H18-01961). Full details on item generation, survey development, and recruitment strategies are published elsewhere (17, 18).

### Sample

Of 6,096 participants in the RESPCCT survey, 309 (6.7%) self-identified as Indigenous to Canada. Due to the smaller sample of participants who identified as Indigenous and completed the measures of interest for this analysis, we were unable to disaggregate the data to support meaningful nation-specific analyses. While this limits the ability to reflect the distinct experiences of First Nations, Inuit, and Métis participants, it also reflects broader structural challenges in achieving adequate representation in larger datasets. Despite this limitation, it remains critical to examine and report these findings, as they provide important insight into patterns of inequity and experiences of care that might otherwise remain obscured.

### Measures

The systematic review and Delphi process that informed how RMC should be measured in the RESPCCT survey identified 17 domains of RMC and its flipside: disrespect & abuse. The current paper includes two outcome measures with items that span several of these domains, including physical and verbal mistreatment, violations of consent, privacy and confidentiality and stigma and discrimination: An 11-item mistreatment index (range 0–11) and a 9-item disrespect index (range 0–9). The scoring of the items that comprise the indices is reported under Tables 1, 2. Respondents who experienced *any mistreatment or any disrespect* during childbirth were coded as 1 and those who checked none of the items were coded as 0. Of 309 Indigenous participants, 230 completed the mistreatment items and 232 the disrespect items.

**Table 1:** Mistreatment Index items, stratified by ethno-racial group.

Item number	During my labour and/or birth, I experienced the following interactions with one or more providers:	Indigenous women (*n* = 230) *n* (%)	Racialized women (*n* = 472) *n* (%)	White women (*n* = 2,868) *n* (%)	*p*	Cramér's V
1	My physical privacy was violated (e.g., being uncovered or having people in the delivery room without my consent).	45 (20.9)	74 (16.6)	459 (16.9)	0.297	0.03
2	My health care provider(s) shouted at or scolded me.	59 (28.0)	68 (16.4)	474 (18.0)	<0.001	0.07
3	My health care provider(s) withheld treatment or forced me to accept treatment that I did not want.	65 (31.0)	82 (20.0)	509 (19.4)	<0.001	0.07
4	My health care provider(s) threatened me.	16 (7.8)	31 (7.7)	154 (5.9)	0.261	0.03
5	Health care provider(s) ignored me, refused my requests for help, or failed to respond to my requests for help in a reasonable amount of time.	72 (34.8)	104 (25.2)	490 (18.7)	<0.001	0.11
6	I experienced physical abuse (including aggressive physical contact, refusal to provide anesthesia for an episiotomy, etc.).	22 (10.8)	37 (9.2)	161 (6.3)	0.007	0.06
7	My private or personal information was shared without my consent.	44 (19.4)	42 (9.3)	174 (6.2)	<0.001	0.13
8	Without my consent, I had surgery that made me unable to have more babies.	5 (2.2)	10 (2.2)	16 (0.6)	<0.001	0.07
9	I was forced to sign legal documents to consent to procedures against my will.	18 (7.9)	12 (2.7)	78 (2.8)	<0.001	0.07
Please answer the questions below about your choices during labour and/or birth.						
10	One or more of my health care providers were physically rough when treating me.	48 (23.2)	62 (15.2)	332 (12.5)	<0.001	0.08
Please describe how many health care providers spoke about or treated you in this way:						
11	Health care providers told me that my baby/babies would have a poor outcome if I did not follow their advice.	76 (36.5)	118 (28.0)	750 (28.3)	0.038	0.05
	Participant checked one or more items above	145 (63.0)	243 (51.5)	1,416 (49.4)	<0.001	0.06

Response options: 1-Family doctor 2-Midwife 3-Nurse 4-Obstetrician 5-Other (e.g., anesthetist, pediatrician, social worker) 6-Not applicable (applies to items 1–6); 1-Always, 2-Often, 3-Sometimes, 4-Never (applies to items 7–9); 1-No, 2-Yes, 3-Not applicable (applies to item 10); 1-All, 2-Some, 3-One, 4-None (applies to item 11).

**Table 2:** Disrespect index items, stratified by ethnoracial group.

Item number	Some people report difficult interactions during labour, birth or after birth. If you have experienced any of the following, please indicate the type of provider you are referring to.	Indigenous women (*n* = 232) *n* (%)	Racialized women (*n* = 467) *n* (%)	White women (*n* = 2,863) *n* (%)	*p*	Cramér's V
1	My health care provider(s) or other staff member(s) made negative comments to me regarding my sexual activity.	16 (7.4)	10 (2.4)	43 (1.6)	<0.001	0.10
2	My health care provider(s) or other staff member(s) made negative comments about my physical appearance (such as my weight, private parts, cleanliness, or other parts of my body)	41 (19.4)	22 (5.4)	206 (7.8)	<0.001	0.11
3	I was mocked by my health care provider(s) or other staff.	42 (19.6)	43 (10.4)	269 (10.3)	<0.001	0.07
4	During my childbirth I felt neglected by my health care provider(s).	84 (39.6)	113 (27.8)	643 (24.5)	<0.001	0.09
5	I was left unattended by my health care provider(s) when I needed care.	79 (37.1)	105 (25.5)	592 (22.5)	<0.001	0.09
6	A health care provider made negative comments regarding my ethnicity, heritage or culture.	17 (8.4)	19 (4.7)	15 (0.6)	<0.001	0.18
	Please answer the questions below about your rights during your labour and/or birth.					
7	My health care provider(s) always, often or sometimes walked in and uncovered me without speaking to me first.	39 (17.2)	51 (11.4)	284 (10.1)	0.003	0.06
8	My health care provider(s) often, sometimes or never asked my permission for a student to do examinations or procedures on me.	129 (58.6)	242 (53.9)	1,131 (41.1)	<0.001	0.12
9	My health care provider(s) often, sometimes or never asked for permission before performing a vaginal examination.	87 (38.3)	132 (29.3)	698 (24.9)	<0.001	0.08
	Participant checked one or more items above	173 (74.6)	323 (69.2)	1,672 (58.4)	<0.001	0.11

Response options: 1-Family doctor 2-Midwife 3-Nurse 4-Obstetrician 5-Other (e.g., anesthetist, pediatrician, social worker) 6-Not applicable (applies to items 1–6); 1-Always, 2-Often, 3-Sometimes, 4-Never (applies to items 7–9).

### Participant characteristics and contextual factors

#### Ethno-racial identity

Ethno-racial identity was self-reported by RESPCCT participants, who indicated their ethnicity or cultural heritage by selecting all applicable categories from a comprehensive list, including Indigenous, racial, and regional identities, as well as options for multi-ethnic, other, or non-response. Participants selecting any category were directed to follow-up questions to provide additional detail, such as specifying Indigenous identity and naming their community or Nation. To analyze respondents who selected multiple categories, an iterative recoding scheme was applied to assign each participant to a single category. Indigenous to Canada responses were prioritized, followed by Black, Asian, Middle Eastern or Hispanic categories, with White assigned only when no other racial category applied. Participants identifying as Indigenous to other countries were categorized according to their other selections. Multi-ethnic responses were reassigned when combined with other categories, while those selecting only “Multi-ethnic” remained in that category.

#### Partner identity

Participants who noted that their partner was involved in care, answered questions about the identity of their partner, including whether the partner is Indigenous or not.

#### Gender and Sexual identity

Gender identity responses, including text-based answers were collapsed into cisgender (majoritized), and gender minoritized (including transgender, non-binary, and two-spirit). Sexual identity responses were categorized into sexual majority (heterosexual/straight) and sexual minority groups (including gay/lesbian, bisexual, queer, two-spirit, asexual, pansexual, heteroflexible, and demisexual).

#### Age

Age at the start of pregnancy was calculated by subtracting the respondent's birth year from the year they first learned they were pregnant.

#### Education

Participants' self-reported education at pregnancy onset was recoded into four categories: some high school, postsecondary or apprenticeship, undergraduate degree, and graduate/professional degree, with “Other” responses excluded.

#### Financial stress

Participants' income was categorized into three levels: more than enough, enough, and not enough to cover monthly expenses, reflecting their perceived ability to meet financial needs.

#### Pre-pregnancy BMI

Pre-pregnancy BMI was calculated based on the weight and height provided by respondents. Pre-pregnancy BMI was then categorized as Normal (18.5–24.9), Overweight (25.0–29.0), and Obese (30.0 and over).

#### Prenatal medical risk

Pregnancy risk was coded by a midwifery student and classified by a senior midwife on the team as low, moderate, or high based on pre-defined survey responses, and open-ended answers about pregnancy complications.

Participants completed several questions about the characteristics of their prenatal and intrapartum healthcare providers, including one question about whether they had any Indigenous provider involved in their prenatal care and a question about who provided intrapartum care. Response options included, midwife, family, physician, obstetrician, and other. Mode of birth was categorized into “Vaginal” (excluding assisted vaginal births) and “Caesarean.”

## Analysis

We report frequencies and proportions for each mistreatment and disrespect item, in addition to the proportion who experienced any mistreatment and disrespect, across three groups: 1) Indigenous, 2) racialized and 3) white participants. To examine significant differences across groups, we calculated *p* values based on Pearson's Chi Square test. To assess the magnitude of difference, we calculated unadjusted relative risks and 95% confidence intervals, derived from generalized linear models (Poisson with Log Link), with the Indigenous group as the reference.

To assess whether Indigenous participants with intersecting characteristics and circumstances reported more or less mistreatment and disrespect, we examined the association between mistreatment and disrespect and 1) whether their partner was Indigenous or not, 2) education level, 3) financial stress, 4) age at the beginning of pregnancy, 5) sexual and gender identity, 6) pre-pregnancy BMI and 7) prenatal medical risk level.

To assess whether any contextual factors among Indigenous participants were associated with more or less mistreatment and disrespect, we examined associations with 1) prenatal provider characteristics, 2) intrapartum provider type, and 3) mode of birth. Place of birth and access to doulas are known to influence perinatal experiences but the subsample of Indigenous participants who had a community birth or support from a doula were too small for robust analysis.

Pearson's Chi Square test was used to calculate *p* values for the associations between mistreatment and disrespect and 1) participant characteristics and 2) contextual factors for the Indigenous group. Effect sizes (Cramér's V) are presented alongside *p* values. We deemed *p* values < 0.001 to be statistically significant (Bonferroni correction, *α* = 0.05/44 chi-square tests ≈ 0.001). Over 6,000 participants from all provinces and territories submitted survey responses describing their experiences with care in Canada for pregnancies that occurred between 2009 and 2022. Not all completed the survey, resulting in variable denominators for the different analyses.

Finally, using as a contextual lens, mistreatment and disrespect items were mapped inductively onto the human rights and reconciliation frameworks that Canada has formally endorsed. Specifically, we aligned the findings with the TRC Call to Action #23, that emphasizes the need to address systemic racism and inequities in healthcare, and restoration of Indigenous health systems (Call to Action 22). These calls for action ensure childbirth practices honor Indigenous traditions, reclaim midwifery practices, and acknowledge the colonial legacy of forced sterilizations, medical evacuations, and culturally unsafe care. These calls are further reinforced by the UN Declaration on the Rights of Indigenous Peoples (UNDRIP): Articles 21 and 22, which recognize the rights of Indigenous peoples to access healthcare services that respect their cultural practices and traditional knowledge. To understand broader implications, we examine our results within the Universal Declaration of Human Rights (UDHR), that uphold fundamental principles of dignity, equality, and access to healthcare and Articles 1, 2, and 25 which affirm the inherent rights to life, and freedom from discrimination. (See Supplemental File 1 for a summary of these frameworks).

## Results

In The RESPCCT study, 6.7% of participants identified as Indigenous, 14.7% as Black, Asian, Middle Eastern, Hispanic/Latine or multi-ethnic and 78.6% as white. Indigenous participants from all Canadian provinces and territories participated in the RESPCCT study. One in three Indigenous participants resided in British Columbia during pregnancy (28.5%), one in five in Manitoba (19.1%) and 17.1% in Ontario. One in two (54.2%) identified as First Nations, 38.2% as Métis and 5.8% as Inuk; and 114 participants provided the name of their community or Nation.

### Experiences of mistreatment and disrespect

Indigenous participants consistently reported higher rates of mistreatment and disrespect compared to the other two groups. For example, 28.0% of Indigenous respondents reported being shouted at or scolded by their healthcare providers, significantly higher than white (18.0%) and BPOC (16.4%) respondents (*p* < 0.001). One in three Indigenous respondents said that health care provider(s) withheld treatment or forced them to accept treatment they did not want, compared to 20.0 % of BPOC and 19.4 % of white respondents (*p* < 0.001). Forced sterilization was significantly more likely to be reported among Indigenous participants compared to the other groups. Other types of mistreatment that were significantly more common among Indigenous women were: healthcare providers failing to respond to requests in a timely manner, physical abuse (including aggressive physical contact, refusal to provide anesthesia for an episiotomy, etc.), health care providers being physically rough with women, being threatened that their baby/babies would have a poor outcome if they did not agree to follow healthcare providers advice, having private or personal information shared without consent and being forced to sign legal documents to consent to procedures they did not want (See Table 1).

Significantly more Indigenous participants (63%) reported any mistreatment compared to 49.4% of white and 51.5% of BPOC respondents (*p* < 0.001) (see Table 1). The relative risk of mistreatment was 0.78 (95% CI: 0.71–0.87) for white compared to Indigenous participants and 0.82 (95% CI: 0.72–0.93) for BPOC compared to Indigenous participants, meaning that both white and BPOC women had a significantly lower risk of mistreatment than Indigenous participants.

Significantly more Indigenous participants (74.6%) reported any disrespect, compared to 58.4 % of white and 69.2 % of BPOC respondents (*p* < 0.001) (see Table 2). The relative risk of disrespect was 0.78 (95% CI: 0.72–0.85) for white compared to Indigenous participants and 0.93 (95% CI: 0.84–1.02) for BPOC compared to Indigenous participants, meaning that white (but not BPOC) women had a significantly lower risk of disrespectful care than Indigenous participants (see Table 2). For example, 8.4 % of Indigenous women said that a health care provider made negative comments regarding their ethnicity, heritage or culture compared to 4.7 % of BPOC and 0.6 % of white participants. Indigenous women were also significantly less likely to be asked for permission/consent to have students present or to have vaginal examinations. They were more likely to be left unattended, feel neglected and overhear negative comments from healthcare providers about their sexual activity or physical appearance.

### Indigenous subgroup analysis

Out of the seven maternal characteristics examined, three were significantly associated with mistreatment and two with disrespectful care among Indigenous participants. (See Table 3). Indigenous women who had a partner involved in care were significantly more likely to experience mistreatment and disrespect *if their partner was also Indigenous*: The likelihood of them experiencing mistreatment increased by 15.4 % and the likelihood of them experiencing disrespectful care increased by 17.1 % (absolute differences). Indigenous women 25 years or younger were significantly more likely to experience mistreatment and disrespect than those over age 25: 18.4 % higher for mistreatment and 17.5 % higher for disrespectful care (absolute differences). Indigenous women with low prenatal medical risk were significantly less likely to report mistreatment, compared to those with moderate or high medical risk.

**Table 3:** Association between participant characteristics and mistreatment and disrespect.

Participant characteristics	Any mistreatment	*p*	Cramér's V	Any disrespect	*p*	Cramér's V
All Indigenous participants	145 (63.0)			173 (74.6)		
Partner is Indigenous		0.035	0.14		0.015	0.14
No	100 (59.2)			118 (69.8)		
Yes	44 (74.6)			53 (86.9)		
Education		0.573	0.10		0.086	0.20
Some high school or high school completed	36 (69.2)			47 (90.4)		
Some postsecondary or apprenticeship completed	47 (61.0)			55 (69.6)		
Undergrad degree	36 (64.3)			42 (75.0)		
Graduate or professional degree	21 (55.3)			24 (63.2)		
Income		0.599	0.07		0.111	0.12
More than enough	37 (61.7)			40 (66.7)		
Enough	66 (60.6)			80 (72.7)		
Income not enough to cover expenses	37 (68.5)			46 (83.6)		
Age at beginning of pregnancy		0.007	0.18		0.003	0.17
25 or younger	56 (74.7)			66 (85.7)		
>25	85 (56.3)			103 (68.2)		
Identified as belonging to gender or sexual minority group		0.229	0.08		0.577	0.04
No	100 (61.3)			159 (77.6)		
Yes	32 (71.1)			57 (83.8)		
Pre-pregnancy BMI		0.918	0.03		0.323	0.09
Normal	50 (62.5)			78 (75.7)		
Overweight	36 (65.5)			53 (79.1)		
Obese	41 (62.1)			76 (84.4)		
Prenatal medical risk		0.030	0.18		0.492	0.08
Low	59 (55.1)			77 (72.0)		
Moderate	39 (73.6)			41 (75.9)		
High	45 (70.3)			52 (80.0)		

Some subgroup estimates are based on small sample sizes and should be interpreted with caution.

Indigenous participants who had midwives as their intrapartum provider had significantly lower rates of mistreatment and disrespect than those who had family physicians or obstetricians, with an effect size of 0.23 for mistreatment and 0.32 for disrespect, and those who had a vaginal birth had significantly lower rates of mistreatment compared to those who gave birth by Cesarean. Women who had an Indigenous provider involved in prenatal care (culture matched care) had lower rates of mistreatment and similar rates of disrespect, compared to women without culture matched care, but the differences were not significant (Table 4).

**Table 4:** Association between contextual factors and mistreatment and disrespect among Indigenous participants.

Contextual factors	Any mistreatment	*p*	Cramér's V	Any disrespect	*p*	Cramér's V
	145 (63.0)			173 (74.6)		
Culture-matched prenatal care^a^		0.140	0.11	0.11	0.703	0.03
No	104 (68.0)			118 (76.6)		
Yes	21 (55.3)			31 (79.5)		
Intrapartum provider was midwife		< 0.001	0.23		< 0.001	0.32
No	126 (68.5)			151 (81.6)		
Yes	19 (41.3)			22 (46.8)		
Mode of birth was:		0.074	0.12		0.314	0.07
Vaginal (excludes assisted vaginal birth)	81 (56.6)			103 (71.0)		
Cesarean	44 (69.8)			49 (77.8)		

Some subgroup estimates are based on small sample sizes and should be interpreted with caution.

a Measured with one item: Did you have any Indigenous care providers during the chosen pregnancy? (1) Yes, I received all of my prenatal care from an Indigenous doctor, midwife, or nurse. (2) Yes, I received some of my prenatal care from an Indigenous doctor, midwife, or nurse. (3) Yes, another care provider involved in my care was Indigenous. (4) No, none of the care providers were Indigenous. (5) I don't know. The first three responses options were combined to indicate culture-matched care and “I don't know” responses were excluded.

## Understanding RESPCCT findings within the context of TRC, UNDRIP and UDHR

The most significant findings point to widespread violations of Indigenous women's core rights. Nearly 40% of respondents reported neglect during childbirth, directly contravening both Article 22 (which affirms special protections for Indigenous women and children) and Article 24 (which affirms the right to the highest attainable standard of health).

Physical and emotional safety were also compromised, reinforcing the depth of systemic disregard in perinatal care: 31.0% reported that treatment was either withheld or forced upon them, raising concerns related to Article 7 of the United Nations Declaration on the Rights of Indigenous Peoples, which affirms the right to security of the person, as well as Articles 3 and 19, which speak to self-determination and free, prior, and informed consent in decisions affecting care. These findings are inconsistent with the Truth and Reconciliation Commission's Calls to Action #22–24, which call for the recognition of Indigenous healing practices, the provision of culturally safe healthcare services, and the elimination of jurisdictional and systemic barriers that undermine Indigenous Peoples' access to respectful and equitable care. Compounding these findings, 34.8% reported being ignored or refused care, and 37.1% were left unattended when in need, suggesting breaches of both safety and ethical standards of care. These experiences reflect concerns aligned with Article 7 and Article 24 of the United Nations Declaration on the Rights of Indigenous Peoples, which affirm the rights to security of the person and access to health services, as well as Article 19, which upholds free, prior, and informed consent in healthcare decision-making. These findings contravene the Truth and Reconciliation Commission's Call to Action #18, which calls for the recognition of health disparities experienced by Indigenous Peoples and the urgent need to address inequities in health outcomes.

What follows are results presented within specific sections of the Truth and Reconciliation Commission Calls (TRC) to Action, United Nations Declaration on the rights of Indigenous People UNDRIP, or the United Declaration of Human Rights (UDHR). Presented in this way, the results must be viewed within Canada's commitment to the United Nations Declaration on the Rights of Indigenous Peoples (UNDRIP), which affirms Indigenous peoples' rights to maintain and practice their cultural traditions (see Table 5).

**Table 5:** Mapping RESPCCT items to UNDRIP and Truth & Reconciliation Calls to Action.

UNDRIP article/Truth & Reconciliation principle	Relevant mistreatment & disrespect items
A) **Article 7**: Right to Life, Liberty, and Security of Person	- My healthcare provider(s) threatened me.
	- I experienced physical abuse (including aggressive physical contact, refusal to provide anesthesia for an episiotomy, etc.).
	- My private or personal information was shared without my consent.
	- Without my consent, I had surgery that made me unable to have more babies.
	- I was forced to sign legal documents to consent to procedures against my will.
	- Health care providers told me that my baby/babies would have a poor outcome if I did not follow their advice.
	- My healthcare provider(s) withheld treatment or forced me to accept treatment that I did not want.
B) **Article 21**: Right to the Improvement of Economic and Social Conditions	- Health care provider(s) ignored me, refused my requests for help, or failed to respond in a reasonable time.
	- I was left unattended by my healthcare provider(s) when I needed care.
C) **Article 22**: Particular Attention to the Rights of Indigenous Women and Children	- During my childbirth I felt neglected by my health care provider(s).
	- My physical privacy was violated (e.g., being uncovered or having people in the delivery room without my consent).
	- My health care provider(s) often, sometimes or never asked my permission for a student to do examinations or procedures on me.
	- My health care provider(s) often, sometimes or never asked for permission before performing a vaginal examination.
D) **Article 24**: Right to the Highest Attainable Standard of Health	- During my childbirth, I felt neglected by my healthcare provider(s).
	- My healthcare provider(s) withheld treatment or forced me to accept treatment that I did not want.
E) **Article 29**: Right to Conservation of Health	
F) **Truth & Reconciliation Call to Action #18**: Health Equity for Indigenous Peoples	- Health care provider(s) ignored me, refused my requests for help, or failed to respond in a reasonable amount of time.
	- My healthcare provider(s) shouted at or scolded me.
	- I experienced physical abuse
	- My health care provider(s) or other staff member(s) made negative comments to me regarding my sexual activity.
	- I was mocked by my health care provider(s) or other staff.
	- My health care provider(s) always, often or sometimes walked in and uncovered me without speaking to me first.
G) **Article 8**: Right Not to be Subjected to Forced Assimilation or Destruction of Culture and **Truth & Reconciliation Call to Action #22**: Recognition of Indigenous Healing Practices	- A healthcare provider(s) made negative comments regarding my ethnicity, heritage, or culture.
H) **Truth & Reconciliation Call to Action #23**: Cultural Safety in Healthcare	- A healthcare provider(s) made negative comments regarding my ethnicity, heritage, or culture.
	- A healthcare provider(s) or other staff member(s) made negative comments about my physical appearance.

Items were mapped to all articles whose protected interest—bodily autonomy, cultural integrity, security of the person—was implicated, with mapping reviewed and approved by the Indigenous lead author and Elde.

### Article 1 & 2 UDRH & Article 2, 9 UNDRIP (rights to be free of discrimination, to be treated with dignity and respect)

The United Nations Declaration on the Rights of Indigenous Peoples (UNDRIP) and the Universal Declaration of Human Rights (UDHR) establish fundamental protections against discrimination and affirm the right to dignity and respect for all individuals, particularly Indigenous peoples. UNDRIP Articles 2 and 9 explicitly prohibit any form of discrimination against Indigenous peoples, whether based on identity, culture, or governance, and affirm their right to belong to an Indigenous community or nation in accordance with their traditions and laws. Similarly, the UDHR Articles 1 and 2 emphasize the inherent dignity and equal rights of all human beings, affirming freedom from discrimination based on race, language, or other distinctions.

In Tables 1, 2, however, we observed significant disparities in rates of mistreatment and disrespect of Indigenous (First Nations, Métis, and Inuit) respondents during childbirth compared to white participants and racialized people (BPOC). The data reveals substantial concerns regarding the treatment of Indigenous people in healthcare settings, with frequent reports of threats, neglect, and violations of cultural rights. Indigenous respondents consistently reported higher rates of mistreatment across multiple dimensions. For instance, 20.9% of Indigenous respondents reported that their physical privacy was violated during childbirth, compared to 16.9% of white and 16.6% of BPOC respondents. Additionally, 28.0% of Indigenous respondents experienced shouting or scolding from their healthcare provider, a significantly higher proportion compared to white (18.0%) and BPOC (16.4%) respondents, while another 19.4% who experienced negative comments about their physical appearance from healthcare providers.

Mistreatment specific to withholding or forcing treatment was reported by 31.0% of Indigenous respondents, compared to 19.4% of white and 20.0% of BPOC respondents. Similarly, 34.8% of Indigenous respondents reported that their healthcare provider ignored them or refused their requests for help, while only 18.7% of white and 25.2% of BPOC respondents reported the same. Furthermore, 8.4% of Indigenous respondents reported receiving negative comments regarding their ethnicity, compared to just 0.6% of white respondents. An alarming 37.1% of Indigenous respondents reported being left unattended when they needed care.

### UNDRIP Article 11, 13 & 31 right to practice, revitalize, develop, protect cultural traditions, Truth & Reconciliation Calls to Action #22: recognition of Indigenous healing practices

The United Nations Declaration on the Rights of Indigenous Peoples (UNDRIP) affirms the rights of Indigenous peoples to express and preserve their culture: Article 11 guarantees the right to practice, protect, and revitalize their cultural traditions and customs, including ceremonies, designs, and artistic expressions: Article 13 underscores the importance of transmitting cultural heritage, traditional knowledge, and expressions, such as oral traditions and languages, to future generations and Article 31 specifically affirms the right to traditional cultural expression. Together with the Truth and Reconciliation Commission's Calls to Action #22 and #23 on the recognition of Indigenous healing practices and need for cultural safety training for health care providers, these provisions affirm cultural expression as central to Indigenous identity and self-determination and as a right that must be respected within healthcare systems.

However, Indigenous women continue to experience a disproportionate burden of mistreatment and disrespect during childbirth. In our study, 8.4% of respondents reported negative comments about their ethnicity, heritage, or culture, which align with other research documenting a lack of respect for Indigenous cultural practices and knowledge, as well as instances of mistreatment and discrimination within health systems (2). Such experiences stand in direct contradiction to the principles articulated in UNDRIP and the Truth and Reconciliation Commission's Calls to Action, highlighting persistent culturally unsafe and racist interactions within healthcare settings. Collectively, this evidence underscores the need for urgent structural changes to ensure equitable, respectful, and culturally safe childbirth experiences for Indigenous women.

In this context, cultural expression and Indigenous embodiment appear not as protected rights, but as sites of surveillance, judgment, and regulation within healthcare encounters. The presence of negative comments directed at Indigenous identity and appearance providers reflects the ongoing operation of colonial power relations in clinical spaces, where Indigenous ways of being are frequently misunderstood, stigmatized, or devalued. These dynamics not only undermine trust in healthcare providers but may also contribute to delayed care-seeking, reduced engagement with services, and poorer health outcomes (19–22). Taken together, these findings suggest that policy commitments to cultural safety have not yet translated into consistent practice at the point of care.

### UNDRIP, Article 7: right to life, liberty, and security of person, and Article 8: right not to be subjected to forced assimilation of culture

Articles 7 and 8 of the United Nations Declaration on the Rights of Indigenous Peoples (UNDRIP) emphasize fundamental protections for both Indigenous communities and individuals. Article 7 affirms their rights to life, liberty, and security, free from violence and actions aimed at eradicating their culture, while article 8 highlights the right to maintain cultural integrity and obligates states to prevent and address actions that dispossess Indigenous peoples of their lands, traditions, or identity through forced assimilation. Together, these articles underscore the necessity of safeguarding Indigenous peoples' cultural and physical well-being.

However, 7.8% of Indigenous respondents reported being threatened by their healthcare provider, while 10.8% experienced physical abuse, including aggressive contact and denial of anesthesia during medical procedures such as episiotomies. Additionally, over a third of Indigenous respondents indicated that healthcare providers either withheld treatment or forced them to undergo treatment without their consent, reflecting serious breaches of their right to security and bodily autonomy. Taken together, these findings reveal a profound disjunction between the protections affirmed in UNDRIP and the lived realities of Indigenous peoples within healthcare settings. Reports of threats, physical abuse, and coerced or withheld treatment constitute serious violations of Indigenous peoples' rights to security, bodily autonomy, and freedom from violence, as articulated in Articles 7 and 8. These experiences cannot be understood as isolated incidents, but rather as manifestations of structural and interpersonal racism embedded within healthcare systems that continue to exert control over Indigenous bodies and decision-making, reflecting the systemic exclusion and devaluation of First Nations health needs documented in Canadian research (1, 3). Research examining cultural competence and safety in Indigenous health contexts highlights how dominant health systems often fail to integrate Indigenous cultural understandings of well-being and instead marginalize or overlook culturally grounded approaches to care, undermining trust and contributing to poorer engagement with services (Lavoie, 2022).

The persistence of such practices signals a failure of health systems to uphold their obligations to prevent harm, protect cultural integrity, and ensure care that is grounded in respect, consent, and dignity. In this context, healthcare environments become sites of ongoing colonial violence, where the rights affirmed in international declarations remain aspirational rather than operationalized. These findings underscore the urgent need for enforceable accountability mechanisms, mandatory anti-racism and trauma-informed training, and community-controlled approaches to care that centre safety, autonomy, and self-determination, as outlined in the TRC Calls to Action (23).

### UNDRIP Article 22: particular attention to the rights of Indigenous women and children and UNDRIP Article 24: right to the highest attainable standard of health

Article 22 of the United Nations Declaration on the Rights of Indigenous Peoples (UNDRIP) emphasizes the protection of Indigenous women, children, and other vulnerable groups, including safeguards against violence, discrimination, and barriers to full and equitable participation in decisions affecting their well-being. Article 24 affirms the right of Indigenous Peoples to the highest attainable standard of physical and mental health and access to culturally appropriate health services without discrimination.

In this context, our findings indicate significant violations of these rights during childbirth. Specifically, 39.6% of Indigenous respondents reported feeling neglected during childbirth, and 34.8% reported being ignored or refused care. In addition, 28.0% reported being shouted at or scolded by healthcare providers, while 20.9% experienced breaches of physical privacy, including being left uncovered without consent. Furthermore, 31.0% reported that treatment was either withheld or imposed without their consent, and 0.8% reported experiencing physical abuse during childbirth. These findings are inconsistent with Articles 22 and 24 of UNDRIP, which require the protection of Indigenous women from discrimination and violence and affirm their right to safe, respectful, and equitable maternity care.

## Discussion

Indigenous respondents (First Nations, Métis, and Inuit) in the current study reported egregious health and human rights violations during childbirth. When compared to racialized and white participants, our study reveals stark disparities in the treatment of Indigenous respondents, reflecting deep-rooted racism within the healthcare system. This is consistent with a recent scoping review of anti-Indigenous racism in Canadian healthcare that found that Indigenous patients frequently experienced differential treatment, including being ignored, treated more slowly, or not believed by clinicians, patterns rooted in racial bias that contribute to poorer care and outcomes for Indigenous peoples (Cooke, 2024). These disparities are deeply concerning considering Canada's commitment to the United Nations Declaration on the Rights of Indigenous Peoples (UNDRIP) and provides evidence for the ongoing normalization and naturalization of anti-Indigenous racism in Canada as the greater context in which healthcare providers live, grow, learn, train and work- showing the multi-dimensional impacts of structural racism.

The higher proportion of Indigenous respondents reporting mistreatment and disrespect underscores ongoing inequities faced by Indigenous Peoples in healthcare settings and reflects broader systemic issues related to discrimination and inequitable access to quality care. These findings are consistent with other studies indicating that Indigenous populations often experience substandard care and disrespect in healthcare environments, further marginalizing them during critical events such as childbirth (8, 24).

The healthcare system's failure to respect Indigenous cultural practices during childbirth also signals a critical gap in upholding Indigenous rights, given that childbirth is a profoundly personal and culturally significant event. Our findings contradict rights-based frameworks, including the United Nations Declaration on the Rights of Indigenous Peoples (UNDRIP), particularly Article 24, which affirms the right to traditional medicines and health practice, and the Truth and Reconciliation Commission Calls to Action, which call for culturally safe care. Rather than reflecting these commitments, the findings point to ongoing breaches of self-determination, cultural continuity, and equitable health services, reinforcing the need to support Indigenous childbirth ceremonies within clinical settings as a matter of rights, not accommodation (25, 26).

The findings also suggest that efforts to address mistreatment in healthcare, particularly in during pregnancy and childbirth, must be aligned with the principles of reconciliation outlined in the Truth and Reconciliation Calls to Action and calls to decolonize our healthcare for Indigenous people, including respecting and incorporating Indigenous culture and healing practices in our health systems. Emerging evidence demonstrates that incorporating Indigenous healing practices and cultural supports into care delivery, such as the involvement of Elders and the creation of space for ceremony, can improve holistic well-being and patient experience, reflecting the commitments articulated in the Truth and Reconciliation Commission Calls to Action and the United Nations Declaration on the Rights of Indigenous Peoples. These findings align with broader research indicating that the inclusion of Indigenous culture in healthcare settings strengthens patients' sense of respect for their identity and may enhance access to and engagement with services, advancing culturally safe and equitable care environments (1, 3, 27–29). This includes integrating culturally safe care, training healthcare providers in antiracism, and ensuring that Indigenous perspectives are included in healthcare policies. The disproportionate rates of mistreatment and disrespect experienced by Indigenous respondents, especially in relation to cultural disrespect, reflect a failure to deliver equitable and respectful care.

Our Indigenous subset analysis showed that young Indigenous women, women with high risk pregnancies and those with Indigenous partners were especially prone to experience mistreatment or disrespectful care. Addressing these disparities requires targeted interventions that prioritize equity in maternity care, and an awareness of the additional burden placed on Indigenous women with intersecting identities and circumstances.

Indigenous women who were attended by midwives during birth reported significantly lower rates of mistreatment and disrespect and culture matched care was linked to lower rates of mistreatment. These results align with previous research (15, 24, 26).

Addressing these disparities requires targeted interventions that prioritize equity in maternity care, particularly for marginalized populations and those undergoing more invasive medical procedures.

### Considerations for future use of indices

The MIST-11 and DISRESPECT-9 indices were composed of items with different response options and stems (language introducing the items) per our Community Steering Council's preference when constructing a larger survey. Future users of the indices are encouraged to harmonize the response options to “Yes and No” and remove the words “always, often or sometimes” from DISRESPCT items 7, 8 & 9. In addition, both indices should be introduced as follows “During my labour and/or birth, I experienced the following interactions with one or more provider” and other stems removed.

## Limitations

Despite using several strategies to recruit under-represented service users (18) a key limitation of this study is the small sample size, which precluded the ability to conduct analyses specific to Indigenous participants. As a result, we were unable to explore differences or nuances within Indigenous communities. While our discussion is framed within Indigenous rights and health frameworks, including the TRC Calls to Action and UNDRIP, we acknowledge that our findings may not fully capture the diversity of experiences across First Nations, Inuit, and Métis peoples. Lastly, the data described in this paper is based on a cross-sectional survey. Combined with the convenience sampling approach, this limits the generalizability of findings.

## Conclusion

UNDRIP and UDHR provide powerful tools to examine Indigenous people's rights in accessing maternity care. Despite Canada being a signatory to both these declarations, we have uncovered significant violations in the form of racism, discrimination, cultural disconnection, and inequitable access to services. Our findings underscore the urgent need to address systemic inequities and uphold Indigenous women's dignity and cultural identity in our healthcare systems.

The solutions are before us: uphold human rights, uphold the rights of Indigenous people, and implement the Truth and Reconciliation Commission (TRC) Calls to Action. TRC Call to Actions 23 and 24, provide a critical roadmap to address these violations, emphasizing the necessity of increasing the number of Indigenous healthcare professionals in the workforce, eliminating racism in healthcare and integrating cultural safety and antiracism training into medical education and practice. Intersecting the Calls to Action with UNDRIP and UDHR, these frameworks provide a strong argument for equitable treatment and the protection of cultural identities, ensuring that individuals and communities are treated with respect and dignity while safeguarding their rights to self-determination and equality within society.

These Calls to Action are not merely suggestions; they are urgent, non-negotiable demands that compel healthcare systems to dismantle entrenched inequities and align with the foundational principles of equity and non-discrimination set forth in international human rights law. They represent a critical pathway to address systemic racism and ensure that Indigenous peoples receive healthcare that is not only equitable but also culturally competent and safe. Failure to act on these Calls risks perpetuating the status quo of discrimination and disparity that violates the very tenets of justice and human dignity enshrined in global frameworks like UNDRIP and the UDHR.

## Data Availability

The datasets presented in this study can be found in online repositories. The names of the repository/repositories and accession number(s) can be found in the article/Supplementary Material.
